# 4-{[5-(4-Chloro­phen­yl)-1-(4-fluoro­phen­yl)-1*H*-pyrazol-3-yl]carbon­yl}-*N*-ethyl­piperazine-1-carboxamide

**DOI:** 10.1107/S1600536811023178

**Published:** 2011-06-18

**Authors:** Tara Shahani, Hoong-Kun Fun, V. Vijayakumar, R. Venkat Ragavan, S. Sarveswari

**Affiliations:** a X-ray Crystallography Unit, School of Physics, Universiti Sains Malaysia, 11800 USM, Penang, Malaysia; b Organic Chemistry Division, School of Advanced Sciences, VIT University, Vellore 632 014, India

## Abstract

The asymmetric unit of the title compound, C_23_H_23_ClFN_5_O_2_, contains two crystallographically independent mol­ecules. In one mol­ecule, the pyrazole ring makes dihedral angles of 43.93 (7) and 35.82 (7)°, respectively, with the fluoro- and chloro-substituted benzene rings, while the corresponding angles in the other mol­ecule are 52.26 (8) and 36.85 (7)°. The piperazine rings adopt chair conformations. In the crystal, adjacent mol­ecules are connected *via* inter­molecular N—H⋯O, C—H⋯F, C—H⋯N and C—H⋯O hydrogen bonds, forming a two-dimensional network parallel to the *bc* plane. The crystal structure is further stabilized by a weak π–π inter­action with a centroid–centroid distance of 3.6610 (8) Å and by C—H⋯π inter­actions.

## Related literature

For our ongoing research on the synthesis of pyrazole derivatives with anti­microbial activity, see: Ragavan *et al.* (2009 ▶, 2010 ▶); Ragavan & Vijayakumar (2011 ▶). For related structures, see: Shahani *et al.* (2009 ▶, 2010**a* ▶,*b* ▶,c* ▶). For bond-length data, see: Allen *et al.* (1987 ▶). For ring conformations, see: Cremer & Pople (1975 ▶).
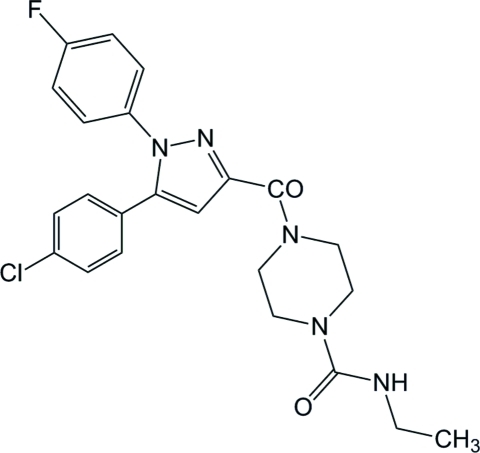


## Experimental

### 

#### Crystal data

C_23_H_23_ClFN_5_O_2_
*M* *_r_* = 455.91
Monoclinic, 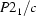

*a* = 25.8566 (6) Å
*b* = 10.0475 (2) Å
*c* = 16.8822 (4) Å
β = 92.525 (1)°
*V* = 4381.64 (17) Å^3^
*Z* = 8
Mo *K*α radiation
μ = 0.21 mm^−1^
*T* = 296 K
0.59 × 0.21 × 0.10 mm

#### Data collection

Bruker SMART APEXII CCD area-detector diffractometer
Absorption correction: multi-scan (*SADABS*; Bruker, 2009 ▶) *T*_min_ = 0.884, *T*_max_ = 0.978
59628 measured reflections
15831 independent reflections
10263 reflections with *I* > 2σ(*I*)
*R*_int_ = 0.038

#### Refinement

*R*[*F* ^2^ > 2σ(*F* ^2^)] = 0.051
*wR*(*F* ^2^) = 0.136
*S* = 1.08
15831 reflections
587 parameters
H atoms treated by a mixture of independent and constrained refinement
Δρ_max_ = 0.47 e Å^−3^
Δρ_min_ = −0.48 e Å^−3^

### 

Data collection: *APEX2* (Bruker, 2009 ▶); cell refinement: *SAINT* (Bruker, 2009 ▶); data reduction: *SAINT*; program(s) used to solve structure: *SHELXTL* (Sheldrick, 2008 ▶); program(s) used to refine structure: *SHELXTL*; molecular graphics: *SHELXTL*; software used to prepare material for publication: *SHELXTL* and *PLATON* (Spek, 2009 ▶).

## Supplementary Material

Crystal structure: contains datablock(s) global, I. DOI: 10.1107/S1600536811023178/is2729sup1.cif

Structure factors: contains datablock(s) I. DOI: 10.1107/S1600536811023178/is2729Isup2.hkl

Supplementary material file. DOI: 10.1107/S1600536811023178/is2729Isup3.cml

Additional supplementary materials:  crystallographic information; 3D view; checkCIF report

## Figures and Tables

**Table 1:** Hydrogen-bond geometry (Å, °) *Cg*1 and *Cg*4 are the centroids of the N4*A*/N5*A*/C9*A*–C11*A* and C18*A*–C23*A* rings, respectively.

*D*—H⋯*A*	*D*—H	H⋯*A*	*D*⋯*A*	*D*—H⋯*A*
N1*B*—H1*NB*⋯O1*A*^i^	0.876 (18)	2.060 (19)	2.9284 (16)	171.1 (16)
N1*A*—H1*NA*⋯O1*B*^ii^	0.905 (18)	2.096 (18)	2.9667 (16)	161.1 (15)
C4*A*—H4*AA*⋯O1*B*^iii^	0.97	2.55	3.4393 (19)	152
C4*A*—H4*AB*⋯O1*B*^ii^	0.97	2.49	3.4466 (17)	168
C6*A*—H6*AA*⋯N4*A*	0.97	2.18	2.9468 (17)	135
C13*A*—H13*A*⋯F1*A*^iv^	0.93	2.52	3.4330 (16)	166
C4*B*—H4*BA*⋯O1*A*^i^	0.97	2.31	3.2757 (17)	175
C22*A*—H22*A*⋯O2*A*^v^	0.93	2.41	3.3167 (18)	164
C22*B*—H22*B*⋯O2*B*^iv^	0.93	2.38	3.1927 (18)	146
C23*A*—H23*A*⋯O2*B*^iii^	0.93	2.47	3.2482 (18)	141
C6*B*—H6*BB*⋯N5*B*	0.97	2.18	2.9505 (17)	136
C7*B*—H7*BA*⋯*Cg*1^vi^	0.97	2.59	3.5216 (16)	162
C2*A*—H2*AB*⋯*Cg*4^vii^	0.97	2.95	3.5831 (15)	124

Symmetry codes: (i) 
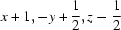
; (ii) 
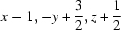
; (iii) 
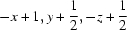
; (iv) 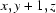; (v) 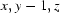; (vi) 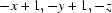; (vii) 
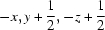
.

**Fig. 1.**
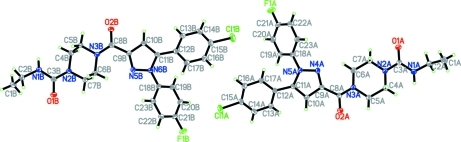
The asymmetric unit of the title compound, showing 30% probability displacement ellipsoids and the atom-numbering scheme.

**Fig. 2.**
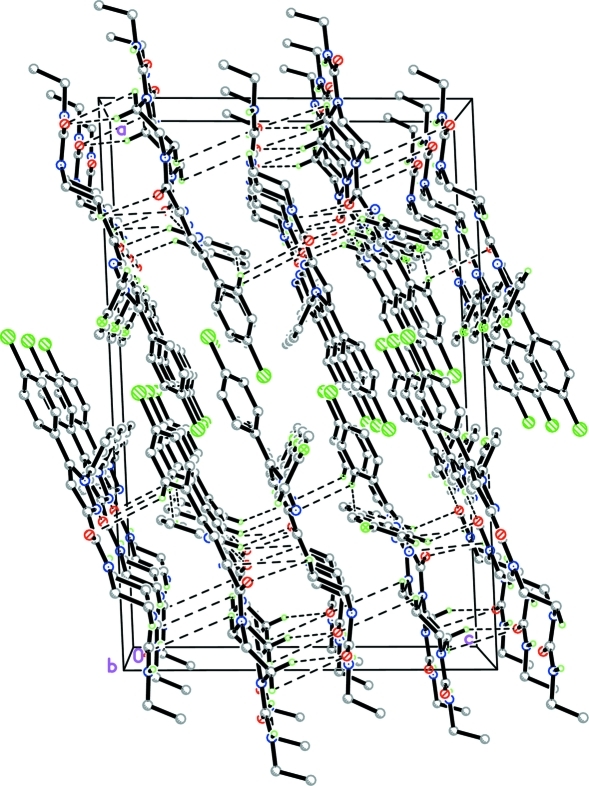
The crystal packing of the title compound, intermolecular hydrogen bonds are shown as dashed lines.

## supplementary crystallographic information

### Comment

As part of our ongoing research aiming on the synthesis of novel pyrazole derivatives as new antimicrobial compounds, herein we report the synthesis of title compound (Ragavan *et al.*, 2009, 2010; Ragavan & Vijayakumar, 2011)

The asymmetric unit of title compound (Fig. 1), contains two crystallographically independent molecules (A & B) in which the pyrazole units are essentially planar, with maximum deviations of 0.008 (1) Å for atom C8A (molecule A) and 0.002 (1) Å for atom C9B (molecule B). The dihedral angle between the pyrazole (N4A/N5A/C9A–C11A)/(N5B/N6B/C9B–C11B) and piperazine rings (N2A/N3A/C4A–C7A)/(N2B/N3B/C4B–C7B) is 1.72 (8) (molecule A) and 22.74 (7)° (molecule B) and that between the fluoro (C18A–C23A)/(C18B–C23B) and chloro-substituted (C12A–C17A)/(C12B–C17B) phenyl rings is 53.97 (7) for molecule A and 55.06 (7)° for molecule B. In each molecule, the piperazine (N2A/N3A/C4A–C7A)/(N2B/N3B/C4B–C7B) ring adopts a chair conformation with puckering parameters (Cremer & Pople, 1975) Θ = 0.5441 (15) Å, θ = 177.80 (16)° and φ = 120 (4)° (molecule A) and Θ = 0.5536 (15) Å, θ = 2.47 (14)° and φ = 329 (4)° (molecule B). The bond lengths (Allen *et al.*, 1987) and angles are within normal ranges and comparable to those closely related structures (Shahani *et al.*, 2009, 2010**a*,b*).

In the crystal structure (Fig. 2), the adjacent molecules are connected *via* intermolecular N1B—H1NB···O1A, N1A—H1NA···O1B, C4A—H4AA···O1B, C4A—H4AB···O1B, C6A—H6AA···N4A, C13A—H13A···F1A, C4B—H4BA···O1A, C22A—H22A···O2A, C22B—H22B···O2B,C23A—H23A···O2B and C6B—H6BB···N5B hydrogen bonds (Table 1), forming a two-dimensional network parallel to the *bc*-plane. Furthermore, the crystal structure is stabilized by weak π–π interactions between pyrazole (N4A/N5A/C9A–C11A)/(N5B/C6B/C9B–C11B) rings [centroid–centroid distance = 3.6610 (8) Å; 1 - *x*, 1/2 + *y*, 1/2 - *z*] and C—H···π interactions, involving *Cg*1 (N4A/N5A/C9A–C11A) and *Cg*4 (C18A–C23A) rings.

### Experimental

The compound has been synthesized using the method available in the literature (Ragavan *et al.*, 2010) and recrystallized with chloroform-methanol 1:1 mixture yielding colourless crystals. *M.p.*: 225.4–226.1 °C

### Refinement

All the H atoms were to C positioned geometrically (C—H = 0.93–0.97 Å) and were refined using a riding model, with *U*_iso_(H) = 1.2 or 1.5 *U*_eq_ (C). The hydrogen atoms bound to N atoms were located in a difference map and were refined freely [N—H = 0.8766 (18)–0.905 (18) Å].

### Crystal data

**Table:**

C_23_H_23_ClFN_5_O_2_	*F*(000) = 1904
*M**_r_* = 455.91	*D*_x_ = 1.382 Mg m^−^^3^
Monoclinic, *P*2_1_/*c*	Mo *K*α radiation, λ = 0.71073 Å
Hall symbol: -P 2ybc	Cell parameters from 9922 reflections
*a* = 25.8566 (6) Å	θ = 2.5–29.8°
*b* = 10.0475 (2) Å	µ = 0.21 mm^−^^1^
*c* = 16.8822 (4) Å	*T* = 296 K
β = 92.525 (1)°	Block, colourless
*V* = 4381.64 (17) Å^3^	0.59 × 0.21 × 0.10 mm
*Z* = 8	

### Data collection

**Table:**

Bruker SMART APEXII CCD area-detector diffractometer	15831 independent reflections
Radiation source: fine-focus sealed tube	10263 reflections with *I* > 2σ(*I*)
graphite	*R*_int_ = 0.038
φ and ω scans	θ_max_ = 32.5°, θ_min_ = 1.6°
Absorption correction: multi-scan (*SADABS*; Bruker, 2009)	*h* = −32→39
*T*_min_ = 0.884, *T*_max_ = 0.978	*k* = −15→14
59628 measured reflections	*l* = −25→24

### Refinement

**Table:**

Refinement on *F*^2^	Primary atom site location: structure-invariant direct methods
Least-squares matrix: full	Secondary atom site location: difference Fourier map
*R*[*F*^2^ > 2σ(*F*^2^)] = 0.051	Hydrogen site location: inferred from neighbouring sites
*wR*(*F*^2^) = 0.136	H atoms treated by a mixture of independent and constrained refinement
*S* = 1.08	*w* = 1/[σ^2^(*F*_o_^2^) + (0.0548*P*)^2^ + 0.7011*P*] where *P* = (*F*_o_^2^ + 2*F*_c_^2^)/3
15831 reflections	(Δ/σ)_max_ = 0.001
587 parameters	Δρ_max_ = 0.47 e Å^−^^3^
0 restraints	Δρ_min_ = −0.48 e Å^−^^3^

### Special details

**Table:**

Geometry. All e.s.d.'s (except the e.s.d. in the dihedral angle between two l.s. planes) are estimated using the full covariance matrix. The cell e.s.d.'s are taken into account individually in the estimation of e.s.d.'s in distances, angles and torsion angles; correlations between e.s.d.'s in cell parameters are only used when they are defined by crystal symmetry. An approximate (isotropic) treatment of cell e.s.d.'s is used for estimating e.s.d.'s involving l.s. planes.
Refinement. Refinement of *F*^2^ against ALL reflections. The weighted *R*-factor *wR* and goodness of fit *S* are based on *F*^2^, conventional *R*-factors *R* are based on *F*, with *F* set to zero for negative *F*^2^. The threshold expression of *F*^2^ > σ(*F*^2^) is used only for calculating *R*-factors(gt) *etc*. and is not relevant to the choice of reflections for refinement. *R*-factors based on *F*^2^ are statistically about twice as large as those based on *F*, and *R*- factors based on ALL data will be even larger.

### Fractional atomic coordinates and isotropic or equivalent isotropic displacement parameters (Å^2^)

**Table:**

	*x*	*y*	*z*	*U*_iso_*/*U*_eq_	
Cl1A	0.485681 (13)	0.77137 (4)	0.09217 (2)	0.02886 (10)	
F1A	0.25759 (4)	0.11751 (8)	0.15316 (6)	0.0340 (2)	
O1A	−0.05009 (4)	0.66568 (10)	0.36382 (7)	0.0243 (2)	
O2A	0.18458 (4)	1.01883 (10)	0.34187 (7)	0.0251 (2)	
N1A	−0.07036 (5)	0.88196 (13)	0.38511 (9)	0.0273 (3)	
N2A	0.01494 (4)	0.81812 (11)	0.36518 (8)	0.0200 (3)	
N3A	0.11718 (4)	0.87662 (11)	0.32283 (7)	0.0192 (2)	
N4A	0.20253 (4)	0.67945 (11)	0.28401 (7)	0.0178 (2)	
N5A	0.24665 (4)	0.64098 (11)	0.24928 (7)	0.0163 (2)	
C1A	−0.14100 (6)	0.85245 (18)	0.47714 (11)	0.0347 (4)	
H1AA	−0.1773	0.8340	0.4792	0.052*	
H1AB	−0.1337	0.9378	0.5006	0.052*	
H1AC	−0.1217	0.7850	0.5059	0.052*	
C2A	−0.12569 (5)	0.85332 (15)	0.39189 (11)	0.0269 (3)	
H2AA	−0.1335	0.7673	0.3682	0.032*	
H2AB	−0.1459	0.9200	0.3628	0.032*	
C3A	−0.03604 (5)	0.78331 (14)	0.37212 (9)	0.0199 (3)	
C4A	0.03840 (5)	0.94481 (14)	0.38959 (9)	0.0213 (3)	
H4AA	0.0538	0.9367	0.4428	0.026*	
H4AB	0.0120	1.0133	0.3901	0.026*	
C5A	0.07963 (5)	0.98407 (14)	0.33283 (9)	0.0214 (3)	
H5AA	0.0633	1.0061	0.2817	0.026*	
H5AB	0.0976	1.0627	0.3529	0.026*	
C6A	0.09284 (5)	0.75057 (14)	0.29914 (10)	0.0241 (3)	
H6AA	0.1190	0.6815	0.2973	0.029*	
H6AB	0.0765	0.7593	0.2466	0.029*	
C7A	0.05269 (5)	0.71207 (14)	0.35762 (10)	0.0226 (3)	
H7AA	0.0352	0.6314	0.3397	0.027*	
H7AB	0.0696	0.6944	0.4090	0.027*	
C8A	0.16832 (5)	0.90795 (14)	0.32237 (9)	0.0184 (3)	
C9A	0.20718 (5)	0.81132 (13)	0.29278 (8)	0.0170 (3)	
C10A	0.25417 (5)	0.85743 (13)	0.26447 (8)	0.0175 (3)	
H10A	0.2663	0.9446	0.2651	0.021*	
C11A	0.27861 (5)	0.74722 (13)	0.23560 (8)	0.0164 (3)	
C12A	0.32866 (5)	0.74362 (13)	0.19775 (8)	0.0163 (3)	
C13A	0.34136 (5)	0.85261 (14)	0.15072 (9)	0.0187 (3)	
H13A	0.3173	0.9203	0.1418	0.022*	
C14A	0.38934 (5)	0.86114 (14)	0.11718 (9)	0.0208 (3)	
H14A	0.3976	0.9338	0.0861	0.025*	
C15A	0.42450 (5)	0.75968 (14)	0.13092 (9)	0.0201 (3)	
C16A	0.41256 (5)	0.64847 (14)	0.17533 (9)	0.0198 (3)	
H16A	0.4364	0.5797	0.1824	0.024*	
C17A	0.36479 (5)	0.64093 (14)	0.20901 (9)	0.0183 (3)	
H17A	0.3567	0.5671	0.2393	0.022*	
C18A	0.25064 (5)	0.50560 (13)	0.22406 (9)	0.0171 (3)	
C19A	0.26649 (5)	0.47606 (14)	0.14866 (9)	0.0196 (3)	
H19A	0.2749	0.5441	0.1141	0.023*	
C20A	0.26974 (5)	0.34438 (14)	0.12526 (9)	0.0232 (3)	
H20A	0.2813	0.3223	0.0756	0.028*	
C21A	0.25541 (6)	0.24670 (14)	0.17733 (10)	0.0234 (3)	
C22A	0.23838 (6)	0.27372 (14)	0.25165 (10)	0.0247 (3)	
H22A	0.2284	0.2055	0.2849	0.030*	
C23A	0.23647 (5)	0.40536 (14)	0.27559 (9)	0.0216 (3)	
H23A	0.2258	0.4266	0.3259	0.026*	
Cl1B	0.422665 (14)	0.26664 (4)	0.22949 (3)	0.03439 (11)	
F1B	0.60085 (4)	0.86517 (8)	0.01124 (6)	0.0326 (2)	
O1B	0.94076 (4)	0.32975 (9)	−0.08019 (6)	0.0230 (2)	
O2B	0.73926 (4)	−0.04416 (9)	0.03639 (6)	0.0237 (2)	
N1B	0.96352 (4)	0.11287 (13)	−0.08865 (8)	0.0213 (3)	
N2B	0.87671 (4)	0.17620 (12)	−0.10070 (8)	0.0222 (3)	
N3B	0.79447 (4)	0.11579 (11)	−0.00106 (8)	0.0199 (3)	
N5B	0.70497 (4)	0.30083 (11)	0.02996 (7)	0.0174 (2)	
N6B	0.65872 (4)	0.34179 (11)	0.05624 (7)	0.0167 (2)	
C1B	1.04218 (6)	0.16926 (15)	−0.16134 (10)	0.0271 (3)	
H1BA	1.0786	0.1863	−0.1533	0.041*	
H1BB	1.0257	0.2451	−0.1861	0.041*	
H1BC	1.0371	0.0927	−0.1948	0.041*	
C2B	1.01871 (5)	0.14349 (15)	−0.08203 (10)	0.0235 (3)	
H2BA	1.0368	0.0697	−0.0562	0.028*	
H2BB	1.0239	0.2214	−0.0487	0.028*	
C3B	0.92797 (5)	0.21163 (14)	−0.08898 (9)	0.0182 (3)	
C4B	0.85644 (5)	0.04105 (14)	−0.09711 (10)	0.0228 (3)	
H4BA	0.8841	−0.0219	−0.1053	0.027*	
H4BB	0.8301	0.0285	−0.1392	0.027*	
C5B	0.83335 (5)	0.01433 (14)	−0.01783 (10)	0.0229 (3)	
H5BA	0.8174	−0.0730	−0.0185	0.028*	
H5BB	0.8605	0.0152	0.0236	0.028*	
C6B	0.81405 (5)	0.25202 (13)	−0.00562 (9)	0.0196 (3)	
H6BA	0.8405	0.2667	0.0361	0.024*	
H6BB	0.7861	0.3146	0.0018	0.024*	
C7B	0.83678 (5)	0.27468 (14)	−0.08610 (9)	0.0220 (3)	
H7BA	0.8095	0.2687	−0.1273	0.026*	
H7BB	0.8516	0.3633	−0.0879	0.026*	
C8B	0.74869 (5)	0.07474 (14)	0.02659 (8)	0.0180 (3)	
C9B	0.70686 (5)	0.17059 (13)	0.04625 (8)	0.0166 (3)	
C10B	0.66187 (5)	0.12823 (13)	0.08232 (8)	0.0174 (3)	
H10B	0.6543	0.0423	0.0988	0.021*	
C11B	0.63125 (5)	0.23920 (13)	0.08848 (8)	0.0161 (3)	
C12B	0.58012 (5)	0.25153 (13)	0.12242 (8)	0.0166 (3)	
C13B	0.54518 (5)	0.14584 (14)	0.11289 (9)	0.0193 (3)	
H13B	0.5544	0.0715	0.0839	0.023*	
C14B	0.49710 (5)	0.14973 (15)	0.14575 (9)	0.0220 (3)	
H14B	0.4742	0.0789	0.1388	0.026*	
C15B	0.48342 (5)	0.26067 (15)	0.18924 (9)	0.0218 (3)	
C16B	0.51731 (5)	0.36627 (14)	0.20055 (9)	0.0208 (3)	
H16B	0.5079	0.4397	0.2302	0.025*	
C17B	0.56554 (5)	0.36181 (14)	0.16724 (8)	0.0186 (3)	
H17B	0.5884	0.4327	0.1748	0.022*	
C18B	0.64400 (5)	0.47767 (13)	0.04455 (8)	0.0173 (3)	
C19B	0.59651 (5)	0.50670 (14)	0.00656 (8)	0.0192 (3)	
H19B	0.5747	0.4383	−0.0111	0.023*	
C20B	0.58200 (6)	0.63773 (14)	−0.00470 (9)	0.0210 (3)	
H20B	0.5504	0.6593	−0.0300	0.025*	
C21B	0.61547 (6)	0.73578 (14)	0.02250 (9)	0.0222 (3)	
C22B	0.66303 (6)	0.71025 (14)	0.05946 (10)	0.0234 (3)	
H22B	0.6848	0.7793	0.0764	0.028*	
C23B	0.67749 (5)	0.57786 (14)	0.07056 (9)	0.0211 (3)	
H23B	0.7093	0.5568	0.0952	0.025*	
H1NB	0.9560 (7)	0.0304 (19)	−0.1012 (11)	0.037 (5)*	
H1NA	−0.0598 (6)	0.9674 (18)	0.3909 (11)	0.031 (5)*	

### Atomic displacement parameters (Å^2^)

**Table:**

	*U*^11^	*U*^22^	*U*^33^	*U*^12^	*U*^13^	*U*^23^
Cl1A	0.01670 (16)	0.0423 (2)	0.0281 (2)	−0.00334 (15)	0.00719 (14)	−0.00694 (17)
F1A	0.0486 (6)	0.0157 (4)	0.0380 (6)	0.0015 (4)	0.0038 (5)	−0.0045 (4)
O1A	0.0210 (5)	0.0150 (5)	0.0373 (7)	−0.0009 (4)	0.0058 (4)	−0.0010 (4)
O2A	0.0237 (5)	0.0162 (5)	0.0360 (7)	−0.0031 (4)	0.0100 (5)	−0.0043 (4)
N1A	0.0181 (6)	0.0151 (6)	0.0493 (9)	−0.0013 (5)	0.0071 (6)	−0.0041 (6)
N2A	0.0178 (5)	0.0130 (6)	0.0295 (7)	0.0008 (4)	0.0052 (5)	−0.0032 (5)
N3A	0.0177 (5)	0.0143 (6)	0.0259 (7)	0.0007 (4)	0.0046 (5)	−0.0026 (5)
N4A	0.0163 (5)	0.0180 (6)	0.0194 (6)	0.0021 (4)	0.0050 (4)	−0.0005 (5)
N5A	0.0158 (5)	0.0139 (5)	0.0196 (6)	0.0010 (4)	0.0039 (4)	0.0001 (5)
C1A	0.0239 (8)	0.0342 (9)	0.0459 (11)	−0.0022 (7)	0.0011 (7)	0.0059 (8)
C2A	0.0165 (6)	0.0198 (7)	0.0449 (10)	0.0001 (5)	0.0057 (6)	−0.0017 (7)
C3A	0.0201 (6)	0.0179 (7)	0.0222 (8)	0.0001 (5)	0.0049 (5)	0.0005 (6)
C4A	0.0206 (6)	0.0147 (7)	0.0291 (8)	−0.0003 (5)	0.0061 (6)	−0.0035 (6)
C5A	0.0207 (6)	0.0153 (7)	0.0287 (8)	0.0025 (5)	0.0060 (6)	0.0013 (6)
C6A	0.0185 (6)	0.0197 (7)	0.0342 (9)	−0.0014 (5)	0.0033 (6)	−0.0093 (6)
C7A	0.0190 (6)	0.0133 (7)	0.0357 (9)	0.0005 (5)	0.0035 (6)	−0.0010 (6)
C8A	0.0193 (6)	0.0171 (7)	0.0191 (7)	−0.0002 (5)	0.0062 (5)	0.0009 (5)
C9A	0.0175 (6)	0.0156 (6)	0.0181 (7)	−0.0006 (5)	0.0029 (5)	−0.0005 (5)
C10A	0.0163 (6)	0.0149 (6)	0.0215 (7)	−0.0006 (5)	0.0013 (5)	0.0000 (5)
C11A	0.0155 (6)	0.0164 (7)	0.0174 (7)	−0.0002 (5)	0.0007 (5)	0.0015 (5)
C12A	0.0139 (6)	0.0180 (7)	0.0169 (7)	−0.0004 (5)	0.0005 (5)	−0.0031 (5)
C13A	0.0165 (6)	0.0181 (7)	0.0214 (7)	0.0007 (5)	0.0004 (5)	0.0001 (5)
C14A	0.0205 (6)	0.0217 (7)	0.0202 (7)	−0.0038 (5)	0.0020 (5)	0.0005 (6)
C15A	0.0156 (6)	0.0258 (8)	0.0192 (7)	−0.0017 (5)	0.0032 (5)	−0.0059 (6)
C16A	0.0168 (6)	0.0221 (7)	0.0201 (7)	0.0038 (5)	−0.0009 (5)	−0.0047 (6)
C17A	0.0181 (6)	0.0176 (7)	0.0190 (7)	0.0004 (5)	0.0001 (5)	−0.0013 (5)
C18A	0.0155 (6)	0.0142 (6)	0.0218 (7)	0.0012 (5)	0.0018 (5)	−0.0007 (5)
C19A	0.0180 (6)	0.0187 (7)	0.0222 (8)	−0.0001 (5)	0.0025 (5)	0.0013 (6)
C20A	0.0246 (7)	0.0226 (8)	0.0226 (8)	0.0016 (6)	0.0039 (6)	−0.0045 (6)
C21A	0.0279 (7)	0.0138 (7)	0.0285 (8)	0.0032 (5)	0.0009 (6)	−0.0029 (6)
C22A	0.0304 (8)	0.0176 (7)	0.0262 (8)	−0.0011 (6)	0.0018 (6)	0.0039 (6)
C23A	0.0254 (7)	0.0195 (7)	0.0201 (7)	0.0016 (6)	0.0032 (6)	0.0002 (6)
Cl1B	0.01845 (17)	0.0472 (3)	0.0382 (2)	0.00217 (16)	0.00826 (16)	0.00418 (19)
F1B	0.0402 (5)	0.0137 (4)	0.0440 (6)	0.0057 (4)	0.0045 (4)	0.0028 (4)
O1B	0.0241 (5)	0.0147 (5)	0.0304 (6)	−0.0019 (4)	0.0043 (4)	−0.0015 (4)
O2B	0.0249 (5)	0.0134 (5)	0.0333 (6)	−0.0005 (4)	0.0083 (4)	0.0037 (4)
N1B	0.0179 (5)	0.0151 (6)	0.0310 (7)	−0.0002 (5)	0.0020 (5)	−0.0007 (5)
N2B	0.0184 (5)	0.0155 (6)	0.0334 (7)	0.0004 (4)	0.0081 (5)	0.0000 (5)
N3B	0.0202 (5)	0.0117 (5)	0.0284 (7)	0.0006 (4)	0.0063 (5)	0.0009 (5)
N5B	0.0153 (5)	0.0164 (6)	0.0209 (6)	0.0015 (4)	0.0039 (4)	0.0007 (5)
N6B	0.0165 (5)	0.0133 (5)	0.0205 (6)	−0.0005 (4)	0.0034 (4)	0.0010 (5)
C1B	0.0245 (7)	0.0225 (8)	0.0346 (9)	−0.0032 (6)	0.0048 (6)	0.0009 (7)
C2B	0.0189 (6)	0.0209 (7)	0.0306 (9)	0.0006 (5)	−0.0021 (6)	−0.0004 (6)
C3B	0.0208 (6)	0.0161 (7)	0.0181 (7)	−0.0004 (5)	0.0038 (5)	0.0009 (5)
C4B	0.0178 (6)	0.0162 (7)	0.0346 (9)	−0.0010 (5)	0.0039 (6)	−0.0057 (6)
C5B	0.0189 (6)	0.0136 (7)	0.0368 (9)	0.0025 (5)	0.0057 (6)	0.0019 (6)
C6B	0.0185 (6)	0.0145 (7)	0.0259 (8)	−0.0006 (5)	0.0026 (6)	−0.0019 (6)
C7B	0.0195 (6)	0.0163 (7)	0.0304 (8)	0.0031 (5)	0.0053 (6)	0.0037 (6)
C8B	0.0194 (6)	0.0170 (7)	0.0176 (7)	−0.0006 (5)	0.0014 (5)	0.0004 (5)
C9B	0.0173 (6)	0.0151 (6)	0.0174 (7)	−0.0002 (5)	0.0014 (5)	0.0000 (5)
C10B	0.0200 (6)	0.0146 (6)	0.0177 (7)	−0.0008 (5)	0.0029 (5)	0.0010 (5)
C11B	0.0183 (6)	0.0145 (6)	0.0156 (7)	−0.0015 (5)	0.0016 (5)	0.0006 (5)
C12B	0.0172 (6)	0.0170 (7)	0.0156 (7)	0.0013 (5)	0.0005 (5)	0.0026 (5)
C13B	0.0222 (7)	0.0165 (7)	0.0191 (7)	−0.0002 (5)	0.0006 (5)	0.0008 (5)
C14B	0.0186 (6)	0.0226 (7)	0.0246 (8)	−0.0038 (5)	−0.0017 (6)	0.0049 (6)
C15B	0.0158 (6)	0.0282 (8)	0.0214 (7)	0.0024 (5)	0.0025 (5)	0.0070 (6)
C16B	0.0217 (6)	0.0216 (7)	0.0191 (7)	0.0047 (5)	0.0020 (5)	0.0008 (6)
C17B	0.0204 (6)	0.0176 (7)	0.0177 (7)	0.0003 (5)	0.0005 (5)	0.0016 (5)
C18B	0.0206 (6)	0.0132 (6)	0.0183 (7)	0.0006 (5)	0.0045 (5)	0.0012 (5)
C19B	0.0225 (6)	0.0175 (7)	0.0176 (7)	0.0011 (5)	0.0030 (5)	−0.0008 (5)
C20B	0.0240 (7)	0.0203 (7)	0.0191 (7)	0.0032 (6)	0.0038 (6)	0.0017 (6)
C21B	0.0301 (8)	0.0131 (7)	0.0241 (8)	0.0042 (5)	0.0080 (6)	0.0013 (6)
C22B	0.0257 (7)	0.0142 (7)	0.0309 (9)	−0.0038 (5)	0.0061 (6)	−0.0014 (6)
C23B	0.0197 (6)	0.0197 (7)	0.0242 (8)	−0.0024 (5)	0.0036 (6)	0.0015 (6)

### Geometric parameters (Å, °)

**Table:**

Cl1A—C15A	1.7422 (14)	Cl1B—C15B	1.7398 (14)
F1A—C21A	1.3626 (16)	F1B—C21B	1.3648 (16)
O1A—C3A	1.2426 (16)	O1B—C3B	1.2391 (16)
O2A—C8A	1.2305 (16)	O2B—C8B	1.2320 (16)
N1A—C3A	1.3546 (18)	N1B—C3B	1.3524 (18)
N1A—C2A	1.4687 (18)	N1B—C2B	1.4594 (18)
N1A—H1NA	0.905 (18)	N1B—H1NB	0.876 (18)
N2A—C3A	1.3737 (17)	N2B—C3B	1.3783 (18)
N2A—C7A	1.4545 (17)	N2B—C4B	1.4578 (18)
N2A—C4A	1.4616 (18)	N2B—C7B	1.4587 (17)
N3A—C8A	1.3594 (17)	N3B—C8B	1.3556 (17)
N3A—C6A	1.4622 (18)	N3B—C6B	1.4626 (17)
N3A—C5A	1.4668 (17)	N3B—C5B	1.4681 (17)
N4A—C9A	1.3380 (17)	N5B—C9B	1.3377 (17)
N4A—N5A	1.3616 (15)	N5B—N6B	1.3577 (15)
N5A—C11A	1.3757 (16)	N6B—C11B	1.3775 (16)
N5A—C18A	1.4304 (17)	N6B—C18B	1.4287 (17)
C1A—C2A	1.510 (2)	C1B—C2B	1.516 (2)
C1A—H1AA	0.9600	C1B—H1BA	0.9600
C1A—H1AB	0.9600	C1B—H1BB	0.9600
C1A—H1AC	0.9600	C1B—H1BC	0.9600
C2A—H2AA	0.9700	C2B—H2BA	0.9700
C2A—H2AB	0.9700	C2B—H2BB	0.9700
C4A—C5A	1.517 (2)	C4B—C5B	1.513 (2)
C4A—H4AA	0.9700	C4B—H4BA	0.9700
C4A—H4AB	0.9700	C4B—H4BB	0.9700
C5A—H5AA	0.9700	C5B—H5BA	0.9700
C5A—H5AB	0.9700	C5B—H5BB	0.9700
C6A—C7A	1.514 (2)	C6B—C7B	1.521 (2)
C6A—H6AA	0.9700	C6B—H6BA	0.9700
C6A—H6AB	0.9700	C6B—H6BB	0.9700
C7A—H7AA	0.9700	C7B—H7BA	0.9700
C7A—H7AB	0.9700	C7B—H7BB	0.9700
C8A—C9A	1.4987 (19)	C8B—C9B	1.4965 (19)
C9A—C10A	1.4039 (18)	C9B—C10B	1.4026 (18)
C10A—C11A	1.3747 (18)	C10B—C11B	1.3742 (18)
C10A—H10A	0.9300	C10B—H10B	0.9300
C11A—C12A	1.4687 (18)	C11B—C12B	1.4691 (19)
C12A—C17A	1.3992 (18)	C12B—C13B	1.3989 (19)
C12A—C13A	1.4003 (19)	C12B—C17B	1.4026 (19)
C13A—C14A	1.3887 (19)	C13B—C14B	1.3840 (19)
C13A—H13A	0.9300	C13B—H13B	0.9300
C14A—C15A	1.379 (2)	C14B—C15B	1.389 (2)
C14A—H14A	0.9300	C14B—H14B	0.9300
C15A—C16A	1.388 (2)	C15B—C16B	1.384 (2)
C16A—C17A	1.3844 (19)	C16B—C17B	1.3909 (19)
C16A—H16A	0.9300	C16B—H16B	0.9300
C17A—H17A	0.9300	C17B—H17B	0.9300
C18A—C19A	1.387 (2)	C18B—C23B	1.3864 (19)
C18A—C23A	1.390 (2)	C18B—C19B	1.3909 (19)
C19A—C20A	1.384 (2)	C19B—C20B	1.3798 (19)
C19A—H19A	0.9300	C19B—H19B	0.9300
C20A—C21A	1.379 (2)	C20B—C21B	1.377 (2)
C20A—H20A	0.9300	C20B—H20B	0.9300
C21A—C22A	1.375 (2)	C21B—C22B	1.378 (2)
C22A—C23A	1.384 (2)	C22B—C23B	1.392 (2)
C22A—H22A	0.9300	C22B—H22B	0.9300
C23A—H23A	0.9300	C23B—H23B	0.9300
			
C3A—N1A—C2A	121.10 (12)	C3B—N1B—C2B	120.51 (12)
C3A—N1A—H1NA	120.9 (10)	C3B—N1B—H1NB	123.3 (12)
C2A—N1A—H1NA	118.0 (10)	C2B—N1B—H1NB	115.2 (11)
C3A—N2A—C7A	118.12 (11)	C3B—N2B—C4B	125.43 (12)
C3A—N2A—C4A	125.84 (11)	C3B—N2B—C7B	118.94 (12)
C7A—N2A—C4A	113.01 (11)	C4B—N2B—C7B	111.50 (11)
C8A—N3A—C6A	127.31 (11)	C8B—N3B—C6B	127.76 (11)
C8A—N3A—C5A	118.63 (11)	C8B—N3B—C5B	118.09 (11)
C6A—N3A—C5A	112.94 (11)	C6B—N3B—C5B	113.46 (11)
C9A—N4A—N5A	104.83 (10)	C9B—N5B—N6B	104.71 (10)
N4A—N5A—C11A	111.89 (10)	N5B—N6B—C11B	112.29 (10)
N4A—N5A—C18A	118.09 (10)	N5B—N6B—C18B	118.53 (10)
C11A—N5A—C18A	129.52 (11)	C11B—N6B—C18B	129.11 (11)
C2A—C1A—H1AA	109.5	C2B—C1B—H1BA	109.5
C2A—C1A—H1AB	109.5	C2B—C1B—H1BB	109.5
H1AA—C1A—H1AB	109.5	H1BA—C1B—H1BB	109.5
C2A—C1A—H1AC	109.5	C2B—C1B—H1BC	109.5
H1AA—C1A—H1AC	109.5	H1BA—C1B—H1BC	109.5
H1AB—C1A—H1AC	109.5	H1BB—C1B—H1BC	109.5
N1A—C2A—C1A	111.88 (14)	N1B—C2B—C1B	113.35 (13)
N1A—C2A—H2AA	109.2	N1B—C2B—H2BA	108.9
C1A—C2A—H2AA	109.2	C1B—C2B—H2BA	108.9
N1A—C2A—H2AB	109.2	N1B—C2B—H2BB	108.9
C1A—C2A—H2AB	109.2	C1B—C2B—H2BB	108.9
H2AA—C2A—H2AB	107.9	H2BA—C2B—H2BB	107.7
O1A—C3A—N1A	121.59 (13)	O1B—C3B—N1B	121.64 (13)
O1A—C3A—N2A	120.62 (12)	O1B—C3B—N2B	120.95 (12)
N1A—C3A—N2A	117.73 (12)	N1B—C3B—N2B	117.40 (12)
N2A—C4A—C5A	110.06 (12)	N2B—C4B—C5B	111.00 (12)
N2A—C4A—H4AA	109.6	N2B—C4B—H4BA	109.4
C5A—C4A—H4AA	109.6	C5B—C4B—H4BA	109.4
N2A—C4A—H4AB	109.6	N2B—C4B—H4BB	109.4
C5A—C4A—H4AB	109.6	C5B—C4B—H4BB	109.4
H4AA—C4A—H4AB	108.2	H4BA—C4B—H4BB	108.0
N3A—C5A—C4A	111.63 (11)	N3B—C5B—C4B	110.34 (12)
N3A—C5A—H5AA	109.3	N3B—C5B—H5BA	109.6
C4A—C5A—H5AA	109.3	C4B—C5B—H5BA	109.6
N3A—C5A—H5AB	109.3	N3B—C5B—H5BB	109.6
C4A—C5A—H5AB	109.3	C4B—C5B—H5BB	109.6
H5AA—C5A—H5AB	108.0	H5BA—C5B—H5BB	108.1
N3A—C6A—C7A	110.01 (12)	N3B—C6B—C7B	109.60 (11)
N3A—C6A—H6AA	109.7	N3B—C6B—H6BA	109.8
C7A—C6A—H6AA	109.7	C7B—C6B—H6BA	109.8
N3A—C6A—H6AB	109.7	N3B—C6B—H6BB	109.8
C7A—C6A—H6AB	109.7	C7B—C6B—H6BB	109.8
H6AA—C6A—H6AB	108.2	H6BA—C6B—H6BB	108.2
N2A—C7A—C6A	110.62 (12)	N2B—C7B—C6B	110.71 (12)
N2A—C7A—H7AA	109.5	N2B—C7B—H7BA	109.5
C6A—C7A—H7AA	109.5	C6B—C7B—H7BA	109.5
N2A—C7A—H7AB	109.5	N2B—C7B—H7BB	109.5
C6A—C7A—H7AB	109.5	C6B—C7B—H7BB	109.5
H7AA—C7A—H7AB	108.1	H7BA—C7B—H7BB	108.1
O2A—C8A—N3A	121.94 (12)	O2B—C8B—N3B	121.49 (12)
O2A—C8A—C9A	116.71 (12)	O2B—C8B—C9B	116.42 (12)
N3A—C8A—C9A	121.23 (12)	N3B—C8B—C9B	122.09 (12)
N4A—C9A—C10A	111.35 (12)	N5B—C9B—C10B	111.27 (11)
N4A—C9A—C8A	128.32 (12)	N5B—C9B—C8B	127.16 (12)
C10A—C9A—C8A	120.18 (12)	C10B—C9B—C8B	121.49 (12)
C11A—C10A—C9A	105.85 (12)	C11B—C10B—C9B	106.16 (12)
C11A—C10A—H10A	127.1	C11B—C10B—H10B	126.9
C9A—C10A—H10A	127.1	C9B—C10B—H10B	126.9
C10A—C11A—N5A	106.06 (11)	C10B—C11B—N6B	105.56 (11)
C10A—C11A—C12A	126.99 (12)	C10B—C11B—C12B	129.01 (12)
N5A—C11A—C12A	126.95 (12)	N6B—C11B—C12B	125.42 (12)
C17A—C12A—C13A	118.81 (12)	C13B—C12B—C17B	118.31 (12)
C17A—C12A—C11A	123.71 (12)	C13B—C12B—C11B	118.60 (12)
C13A—C12A—C11A	117.43 (12)	C17B—C12B—C11B	123.02 (12)
C14A—C13A—C12A	121.02 (13)	C14B—C13B—C12B	121.29 (13)
C14A—C13A—H13A	119.5	C14B—C13B—H13B	119.4
C12A—C13A—H13A	119.5	C12B—C13B—H13B	119.4
C15A—C14A—C13A	118.70 (13)	C13B—C14B—C15B	119.26 (13)
C15A—C14A—H14A	120.7	C13B—C14B—H14B	120.4
C13A—C14A—H14A	120.7	C15B—C14B—H14B	120.4
C14A—C15A—C16A	121.69 (13)	C16B—C15B—C14B	120.90 (13)
C14A—C15A—Cl1A	119.34 (11)	C16B—C15B—Cl1B	119.79 (11)
C16A—C15A—Cl1A	118.97 (11)	C14B—C15B—Cl1B	119.30 (11)
C17A—C16A—C15A	119.32 (13)	C15B—C16B—C17B	119.54 (13)
C17A—C16A—H16A	120.3	C15B—C16B—H16B	120.2
C15A—C16A—H16A	120.3	C17B—C16B—H16B	120.2
C16A—C17A—C12A	120.42 (13)	C16B—C17B—C12B	120.70 (13)
C16A—C17A—H17A	119.8	C16B—C17B—H17B	119.6
C12A—C17A—H17A	119.8	C12B—C17B—H17B	119.6
C19A—C18A—C23A	121.06 (13)	C23B—C18B—C19B	121.34 (13)
C19A—C18A—N5A	120.32 (12)	C23B—C18B—N6B	119.42 (12)
C23A—C18A—N5A	118.57 (13)	C19B—C18B—N6B	119.24 (12)
C20A—C19A—C18A	119.38 (13)	C20B—C19B—C18B	119.52 (13)
C20A—C19A—H19A	120.3	C20B—C19B—H19B	120.2
C18A—C19A—H19A	120.3	C18B—C19B—H19B	120.2
C21A—C20A—C19A	118.48 (14)	C21B—C20B—C19B	118.27 (13)
C21A—C20A—H20A	120.8	C21B—C20B—H20B	120.9
C19A—C20A—H20A	120.8	C19B—C20B—H20B	120.9
F1A—C21A—C22A	118.73 (13)	F1B—C21B—C20B	117.97 (13)
F1A—C21A—C20A	118.11 (14)	F1B—C21B—C22B	118.45 (13)
C22A—C21A—C20A	123.16 (13)	C20B—C21B—C22B	123.57 (13)
C21A—C22A—C23A	118.17 (14)	C21B—C22B—C23B	117.88 (13)
C21A—C22A—H22A	120.9	C21B—C22B—H22B	121.1
C23A—C22A—H22A	120.9	C23B—C22B—H22B	121.1
C22A—C23A—C18A	119.71 (14)	C18B—C23B—C22B	119.40 (13)
C22A—C23A—H23A	120.1	C18B—C23B—H23B	120.3
C18A—C23A—H23A	120.1	C22B—C23B—H23B	120.3
			
C9A—N4A—N5A—C11A	−0.53 (15)	C9B—N5B—N6B—C11B	−0.35 (15)
C9A—N4A—N5A—C18A	−173.20 (12)	C9B—N5B—N6B—C18B	−177.66 (12)
C3A—N1A—C2A—C1A	101.53 (17)	C3B—N1B—C2B—C1B	−87.69 (17)
C2A—N1A—C3A—O1A	1.5 (2)	C2B—N1B—C3B—O1B	−3.3 (2)
C2A—N1A—C3A—N2A	178.95 (14)	C2B—N1B—C3B—N2B	175.78 (13)
C7A—N2A—C3A—O1A	−7.4 (2)	C4B—N2B—C3B—O1B	−168.01 (14)
C4A—N2A—C3A—O1A	−166.25 (14)	C7B—N2B—C3B—O1B	−12.9 (2)
C7A—N2A—C3A—N1A	175.14 (14)	C4B—N2B—C3B—N1B	12.9 (2)
C4A—N2A—C3A—N1A	16.3 (2)	C7B—N2B—C3B—N1B	168.07 (13)
C3A—N2A—C4A—C5A	−144.92 (14)	C3B—N2B—C4B—C5B	99.49 (16)
C7A—N2A—C4A—C5A	55.31 (16)	C7B—N2B—C4B—C5B	−57.23 (16)
C8A—N3A—C5A—C4A	−137.14 (13)	C8B—N3B—C5B—C4B	134.45 (13)
C6A—N3A—C5A—C4A	54.07 (17)	C6B—N3B—C5B—C4B	−54.31 (16)
N2A—C4A—C5A—N3A	−52.52 (16)	N2B—C4B—C5B—N3B	54.13 (16)
C8A—N3A—C6A—C7A	137.43 (14)	C8B—N3B—C6B—C7B	−134.86 (14)
C5A—N3A—C6A—C7A	−54.96 (16)	C5B—N3B—C6B—C7B	54.92 (16)
C3A—N2A—C7A—C6A	141.13 (14)	C3B—N2B—C7B—C6B	−100.30 (15)
C4A—N2A—C7A—C6A	−57.39 (16)	C4B—N2B—C7B—C6B	58.10 (16)
N3A—C6A—C7A—N2A	55.63 (16)	N3B—C6B—C7B—N2B	−55.80 (15)
C6A—N3A—C8A—O2A	178.21 (14)	C6B—N3B—C8B—O2B	−170.97 (14)
C5A—N3A—C8A—O2A	11.2 (2)	C5B—N3B—C8B—O2B	−1.1 (2)
C6A—N3A—C8A—C9A	2.3 (2)	C6B—N3B—C8B—C9B	9.5 (2)
C5A—N3A—C8A—C9A	−164.64 (13)	C5B—N3B—C8B—C9B	179.32 (13)
N5A—N4A—C9A—C10A	−0.47 (15)	N6B—N5B—C9B—C10B	0.39 (15)
N5A—N4A—C9A—C8A	175.10 (14)	N6B—N5B—C9B—C8B	177.27 (13)
O2A—C8A—C9A—N4A	164.53 (14)	O2B—C8B—C9B—N5B	−169.57 (14)
N3A—C8A—C9A—N4A	−19.4 (2)	N3B—C8B—C9B—N5B	10.0 (2)
O2A—C8A—C9A—C10A	−20.3 (2)	O2B—C8B—C9B—C10B	7.0 (2)
N3A—C8A—C9A—C10A	155.83 (14)	N3B—C8B—C9B—C10B	−173.42 (13)
N4A—C9A—C10A—C11A	1.27 (16)	N5B—C9B—C10B—C11B	−0.30 (16)
C8A—C9A—C10A—C11A	−174.71 (13)	C8B—C9B—C10B—C11B	−177.38 (12)
C9A—C10A—C11A—N5A	−1.50 (15)	C9B—C10B—C11B—N6B	0.07 (15)
C9A—C10A—C11A—C12A	178.44 (13)	C9B—C10B—C11B—C12B	−179.32 (14)
N4A—N5A—C11A—C10A	1.31 (15)	N5B—N6B—C11B—C10B	0.17 (16)
C18A—N5A—C11A—C10A	172.92 (13)	C18B—N6B—C11B—C10B	177.12 (13)
N4A—N5A—C11A—C12A	−178.62 (13)	N5B—N6B—C11B—C12B	179.59 (13)
C18A—N5A—C11A—C12A	−7.0 (2)	C18B—N6B—C11B—C12B	−3.5 (2)
C10A—C11A—C12A—C17A	142.40 (15)	C10B—C11B—C12B—C13B	−35.6 (2)
N5A—C11A—C12A—C17A	−37.7 (2)	N6B—C11B—C12B—C13B	145.11 (14)
C10A—C11A—C12A—C13A	−34.7 (2)	C10B—C11B—C12B—C17B	141.27 (15)
N5A—C11A—C12A—C13A	145.25 (14)	N6B—C11B—C12B—C17B	−38.0 (2)
C17A—C12A—C13A—C14A	−1.6 (2)	C17B—C12B—C13B—C14B	0.8 (2)
C11A—C12A—C13A—C14A	175.65 (13)	C11B—C12B—C13B—C14B	177.84 (13)
C12A—C13A—C14A—C15A	0.1 (2)	C12B—C13B—C14B—C15B	−0.2 (2)
C13A—C14A—C15A—C16A	1.8 (2)	C13B—C14B—C15B—C16B	−0.5 (2)
C13A—C14A—C15A—Cl1A	−177.81 (11)	C13B—C14B—C15B—Cl1B	179.17 (11)
C14A—C15A—C16A—C17A	−2.2 (2)	C14B—C15B—C16B—C17B	0.6 (2)
Cl1A—C15A—C16A—C17A	177.41 (11)	Cl1B—C15B—C16B—C17B	−179.05 (11)
C15A—C16A—C17A—C12A	0.7 (2)	C15B—C16B—C17B—C12B	0.0 (2)
C13A—C12A—C17A—C16A	1.2 (2)	C13B—C12B—C17B—C16B	−0.7 (2)
C11A—C12A—C17A—C16A	−175.88 (13)	C11B—C12B—C17B—C16B	−177.58 (13)
N4A—N5A—C18A—C19A	131.60 (13)	N5B—N6B—C18B—C23B	−53.26 (18)
C11A—N5A—C18A—C19A	−39.6 (2)	C11B—N6B—C18B—C23B	129.96 (15)
N4A—N5A—C18A—C23A	−46.05 (17)	N5B—N6B—C18B—C19B	126.13 (14)
C11A—N5A—C18A—C23A	142.78 (14)	C11B—N6B—C18B—C19B	−50.7 (2)
C23A—C18A—C19A—C20A	−2.0 (2)	C23B—C18B—C19B—C20B	−0.8 (2)
N5A—C18A—C19A—C20A	−179.56 (12)	N6B—C18B—C19B—C20B	179.81 (12)
C18A—C19A—C20A—C21A	2.0 (2)	C18B—C19B—C20B—C21B	−0.1 (2)
C19A—C20A—C21A—F1A	178.53 (13)	C19B—C20B—C21B—F1B	−179.93 (13)
C19A—C20A—C21A—C22A	−0.5 (2)	C19B—C20B—C21B—C22B	0.9 (2)
F1A—C21A—C22A—C23A	179.84 (13)	F1B—C21B—C22B—C23B	−179.99 (13)
C20A—C21A—C22A—C23A	−1.2 (2)	C20B—C21B—C22B—C23B	−0.8 (2)
C21A—C22A—C23A—C18A	1.2 (2)	C19B—C18B—C23B—C22B	0.9 (2)
C19A—C18A—C23A—C22A	0.3 (2)	N6B—C18B—C23B—C22B	−179.73 (13)
N5A—C18A—C23A—C22A	177.93 (13)	C21B—C22B—C23B—C18B	−0.1 (2)

### Hydrogen-bond geometry (Å, °)

**Table:**

*Cg*1 and *Cg*4 are the centroids of the N4A/N5A/C9A–C11A and C18A–C23A rings, respectively.

**Table:**

*D*—H···*A*	*D*—H	H···*A*	*D*···*A*	*D*—H···*A*
N1B—H1NB···O1A^i^	0.876 (18)	2.060 (19)	2.9284 (16)	171.1 (16)
N1A—H1NA···O1B^ii^	0.905 (18)	2.096 (18)	2.9667 (16)	161.1 (15)
C4A—H4AA···O1B^iii^	0.97	2.55	3.4393 (19)	152
C4A—H4AB···O1B^ii^	0.97	2.49	3.4466 (17)	168
C6A—H6AA···N4A	0.97	2.18	2.9468 (17)	135
C13A—H13A···F1A^iv^	0.93	2.52	3.4330 (16)	166
C4B—H4BA···O1A^i^	0.97	2.31	3.2757 (17)	175
C22A—H22A···O2A^v^	0.93	2.41	3.3167 (18)	164
C22B—H22B···O2B^iv^	0.93	2.38	3.1927 (18)	146
C23A—H23A···O2B^iii^	0.93	2.47	3.2482 (18)	141
C6B—H6BB···N5B	0.97	2.18	2.9505 (17)	136
C7B—H7BA···Cg1^vi^	0.97	2.59	3.5216 (16)	162
C2A—H2AB···Cg4^vii^	0.97	2.95	3.5831 (15)	124

Symmetry codes: (i) *x*+1, −*y*+1/2, *z*−1/2; (ii) *x*−1, −*y*+3/2, *z*+1/2; (iii) −*x*+1, *y*+1/2, −*z*+1/2; (iv) *x*, *y*+1, *z*; (v) *x*, *y*−1, *z*; (vi) −*x*+1, −*y*+1, −*z*; (vii) −*x*, *y*+1/2, −*z*+1/2.
